# Nurse practitioner consultations in primary health care: patient, carer, and nurse practitioner qualitative interpretations of communication processes

**DOI:** 10.1017/S1463423618000798

**Published:** 2018-10-31

**Authors:** Julian Barratt, Nicola Thomas

**Affiliations:** 1 Head of Community Nursing, Institute of Health, Faculty of Education, Health and Wellbeing, University of Wolverhampton, Wolverhampton, UK; 2 Associate Professor in Kidney Care, School of Health and Social Care, London South Bank University, London, UK

**Keywords:** advanced clinical practice, consultations, health communication, nurse practitioners, qualitative research, social interactions

## Abstract

**Aim:**

To advance understanding of the discrete nature of the communication processes and social interactions occurring in nurse practitioner consultations.

**Background:**

Preceding qualitative investigations of nurse practitioner consultations have, when conducting interviews with participants, often exclusively sampled either nurse practitioners or patients. Furthermore, previous qualitative studies of the nature of nurse practitioner consultations have not typically also sampled carers attending with patients for nurse practitioner consultations. Accordingly this study was developed, in part, to address this exclusivity of sampling in qualitative research of nurse practitioner consultations by developing an inclusive sample of patient, carer and nurse practitioner participants of nurse practitioner consultations, so as to conjointly develop an understanding of the multiple perceptions of those participants of communication processes occurring in nurse practitioner consultations.

**Methods:**

Qualitative component of a larger mixed methods case study of communication processes and social interactions in nurse practitioner consultations, utilising individual semi-structured interviews with the patient (*n* = 9), carer (*n* = 2) and nurse practitioner (*n* = 3) participants of video-recorded consultations derived from a nurse practitioner-led general practice clinic. Interview transcripts were initially analysed via an emergent thematic analysis, followed up by computer-assisted qualitative data analysis with *NVivo 9*.

**Findings:**

The participants’ perceptions of nurse practitioner consultation communication processes and social interactions were represented through six themes: Consulting style of nurse practitioners; Nurse practitioner – GP comparisons; Lifeworld content or lifeworld style; Nurse practitioner role ambiguity; Creating the impression of time and Expectations for safety netting. The findings identify a need for policy makers to address a perceived ambiguity of the nature of the nurse practitioner role amongst patients and carers. The benefits of nurse practitioners using personable, everyday lifeworld styles of communication for optimising interactions, sharing clinical reasoning and conveying a sense of having time for patients and carers in consultations are also identified.

## Introduction

Nurses working in advanced clinical roles such as advanced nurse practitioners or advanced clinical practitioners are increasingly engaging in clinical consultation activities once mostly associated with medical doctors, such as clinical reasoning to establish differential diagnoses for patients’ presenting medical problems and prescribing medicines (Health Education England, 2017; Barratt, 2018). Currently available qualitative studies of participants’ experiences of nurse practitioner consultations have commonly noted nurse practitioners communicate with their patients in a ‘hybrid’ style, combining objective analysis of biomedical information together with discussion of issues from their everyday lifeworld (Brykczynski, 1989; Johnson, 1993; Kleiman, 2004; Barratt, 2005; Seale *et al*., 2005, 2006; Williams and Jones, 2006; Defibaugh, 2014a, 2014b; Bentley *et al*., 2016). In this context the term ‘lifeworld’ represents the subjectivities of people’s everyday life experiences being integrated in clinical consultations (Thomas, 2010). Other qualitative studies of nurse practitioner consultations have focused on patients’ expectations of consulting with nurse practitioners (Redsell *et al*., 2007a; 2007b), or patients’ perceptions of clinical uncertainty in nurse practitioner consultations (Barnes *et al*., 2004).

However, those qualitative investigations of nurse practitioner consultation have, when conducting interviews with participants, often exclusively sampled either nurse practitioners (Barnes *et al*., 2004; Kleiman, 2004; Barratt, 2005), or patients (Williams and Jones, 2006; Redsell *et al*., 2007a, 2007b). Furthermore, currently available qualitative studies of the nature of nurse practitioner consultations have not typically also sampled carers attending with patients for nurse practitioner consultations, although a qualitative study of nurse prescribing in a dementia clinic has explored patients’, carers’ and staff experiences of nurse prescribing, but the study did not include any nurse practitioners (Grant *et al*., 2007; Page *et al*., 2008). Accordingly, the study presented in this paper inclusively sampled patient, carer and nurse practitioner participants of nurse practitioner consultations, so as to conjointly develop an understanding of the multiple perceptions of those participants of communication processes in nurse practitioner consultations.

## Study design, aim and research questions

This report presents the findings of the qualitative component of a larger mixed methods case study of communication processes and social interactions in nurse practitioner consultations (Barratt, 2016). The convergent parallel mixed methods case study (Creswell, 2014) was intended to concomitantly scrutinise the communication processes, social interactions and measured outcomes of nurse practitioner consultations using three parallel strands of inquiry: video recordings of nurse practitioner consultations; a validated questionnaire measuring patient expectations, patient satisfaction and patient enablement and also semi-structured individual interviews with selected patient, carer and nurse practitioner participants of the video-recorded consultations. The detailed results of the video recorded and questionnaire components of the study are presented elsewhere in *Primary Health Research & Development* (Barratt and Thomas, 2018a, 2018b); this paper focuses on reporting the qualitative dimension of the mixed methods case study.

This study aimed to advance understanding of the discrete nature of the communication processes and social interactions occurring in nurse practitioner consultation. The research questions in the qualitative section of the study were:

- What are patients’, carers’ and nurse practitioners perceptions of interaction styles, inclusion of lifeworld information and social status of the nurse practitioner role in nurse practitioner consultations?
- What are patients’, carers’ and nurse practitioners’ impressions of the time length duration of nurse practitioner consultations?
- What are patients’ and carer’ expectations of consulting with nurse practitioners?

## Methods

For the interviews, a semi-structured interview technique was chosen to allow respondents their say on the topic of enquiry (Dearnley, 2005). Semi-structured interviews also enabled exploration of information relevant to the study’s aim and research questions and any subsequent areas of mutual interest that emerged (Houghton *et al*., 2013). For all of the patient/carer interviews, the same semi-structured schedule was used with flexible variations in the interview content derived from the subsequent interview interactions with the participants. The interview schedule was developed in relation to the stated aim and research questions of the study, seeking to elicit patients’/carers’ views on consulting with a nurse practitioner, including everyday discussing lifeworld information. As with the patient/carer interviews schedule a nurse practitioner interview schedule was similarly developed in relation to stated aim and research questions of the study, seeking to elicit their views on consulting with patients as a nurse practitioner.

### Setting and participants

The study’s setting in primary health care was a general practice clinic in a United Kingdom city, where the patients mostly have consultations with nurse practitioners. A convenience sample of patients, carers and nurse practitioners who had participated in preceding video-recorded consultations were also asked to participate in semi-structured interviews. Nine patients, two carers (mothers of child patients) and three nurse practitioners agreed to participate in the interviews.

### Data collection

The majority of data collection for the whole mixed methods study took place over a 14-month period starting in September 2011 and finishing in November 2012. The ensuing data analysis was completed between 2012 and 2016. To enhance the credibility of the study a supplemental period of data collection was completed in October 2016, involving presenting the findings to the nurse practitioner participants, to facilitate respondent validation for reflectively discussing the study’s findings, with the outcomes of that discussion being integrated in the final analyses of the study (Birt *et al*., 2016).

### Data analysis

All interviews were audio recorded and then fully transcribed as the first part of the data analysis process. The initial stages of data analysis comprised an emergent thematic analysis of the interview data involving an iterative, interlinked process of data familiarisation, data reduction, data display and summarising. Miles and Huberman’s (1994) approach was chosen for guiding the initial stages of analysis because their analytic techniques are recommended for putting collected data in case studies in order prior to detailed analysis (Yin, 2014; Houghton *et al*., 2017). Once the emergent thematic analysis had been completed computer-assisted qualitative data analysis (CAQDA) then provided the subsequent determinant approach to the data analysis process for the interviews via the use *of NVivo 9* software (Bazeley and Jackson, 2013). It has been noted that there should be no ‘false dichotomy between tool and process’ in CAQDA and that software such as *NVivo* should be viewed as having a complete analytical capability which encompasses both how the analysis is completed (process) and what it is completed with (tool) (Johnston, 2006, p. 381). Furthermore, CAQDA with NVivo has been used successfully in other mixed methods case studies of nurse practitioners (Sangster-Gormley, 2013; Sangster-Gormley *et al*. 2015).

Further details of the components of the steps of the *NVivo* data analysis process and their practical implementation in the study are presented in Table 1.

**Table 1:** Steps of NVivo guided thematic analysis (Bazeley and Jackson, 2013)

*NVivo* analysis steps	Description of qualitative data analysis process implemented in the study
1. Initial coding	Detecting initial codes in the emergent thematic analysis.
2. Identifying and naming codes	Identifying further codes from the data in a structured style, including building on and integrating the initial emergent thematic analysis.
3. Storing codes in nodes in a structured system	Sorting and storing similarly related codes in coding nodes.
4. Comparative coding analysis	Comparative visual analysis of coding nodes with charts, or graphs, or treemaps generated in *NVivo*.
5. Exploring coding node relationships	Exploring coding nodes relationships via modelling of nodes.
6. Conceptualising and aggregating coding nodes	Grouping together coding nodes which are conceptually similar in a hierarchical sequence of child and parent nodes.

### Trustworthiness

Baillie’s (2015) recommendations for promoting scientific rigour in qualitative research enable consideration of the trustworthiness of the study in relation to credibility, transferability, dependability and confirmability. The study is credible as it was based on verbatim transcriptions of reflections on real-life nurse practitioner consultations (MacLean *et al*., 2004), and thus the findings are also transferable to other similar primary health care clinics. The consistent use of interview schedules with participants enhanced the dependability of the findings, and the confirmability of the interpretation of the findings is enriched by the researcher’s nursing background, which is as a nurse practitioner in primary health care, conducting consultations similar to those investigated in this study.

### Ethical considerations

Approval for the study was given by both University and National Health Service research ethics committees. After review, there were no conditions of approval set specifically for the qualitative component of the study being reported in this paper. Local research governance approval was also obtained for conducting research in the selected general practice clinic.

## Findings

Five of the 11 patient/carer post-consultation interviews were face-to-face interviews conducted at general practice clinic, and six of them were telephone interviews. All of those interviews took place within one to two days of their video recorded consultation being recorded. The mean duration of the patient/carer interviews was 9.6 minutes (range 5.09–15.02 minutes). The age range of the patient participants was 41–72 years old. All of the patient/carer participants were White. Three of the patient participants were male and the eight other patient/carer participants were female. Eight of the participants had attended for same day appointments and three had attended for pre-booked appointments. The overview details of the interview participants are displayed in Table 2. The three interviews (one each) with the nurse practitioner participants were all face-to-face individual interviews conducted at the general practice clinic. The mean duration of the nurse practitioner interviews was 41.8 minutes (range 34.5–46.1 minutes). All of the nurse practitioners were women, and had completed university-based accredited education as nurse practitioners, and were recorded with the Nursing and Midwifery Council as nurse independent prescribers.

**Table 2:** Details of the patient/carer interview participants

Patient/carer notation^a^	Patient/carer ethnicity and ages	Consultation appointment type/reason for attendance
Patient 1.3	White British male, 62 years old	*Same day*/Medication request, tiredness
Patient 1.5	White British male, 55 years old	*Same day*/Earache
Patient 1.10	White British female, 68 years old	*Same day*/Skin lesion, toe problems
Patient 2.2	White British male, 72 years old	*Pre-booked*/Swollen eyelid
Patient 2.4	White British female, 41 years old	*Pre-booked*/Breast concerns, anxiety, back pain
Patient 2.8	White British female, 62 years old	*Same day*/Dizziness, hyperlipidaemia
Mother of child patient 2.9	White Italian mother of a 1-year-old child	*Same day*/Fever
Mother of child patient 2.10	White British mother of a 9 months old infant	*Same day*/Oral candida
Patient 3.5	White British female, 59 years old	*Same day*/Infected sebaceous cyst
Patient 3.6	White British female, 51 years old	*Pre booked*/Hypertension review, skin lesion
Patient 3.10	White British female, 72 years old	*Same day*/Back pain post-cystoscopy

^a^The interviewees of nurse practitioner 1 have been notated as: patient 1.3; patient 1.5; and patient 1.10. The interviewees of nurse practitioner 2 have been notated as: patient 2.2; patient 2.4; patient 2.8; mother of child patient 2.9; and mother of child patient 2.10. The interviewees of nurse practitioner 3 have been notated as: patient 3.5; patient 3.6; and patient 3.10.

### Data analysis themes

**Figure 1:**
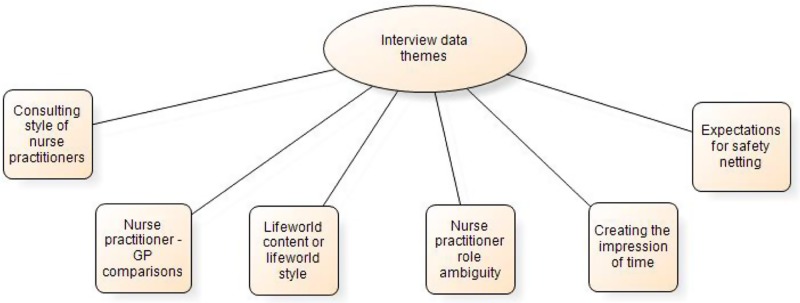
Main themes arising from interview data analysis

At the end of the qualitative data analysis processes, six main themes arising from the interview data were identified. The theme titles are summarily presented in Figure 1.

### Consulting style of nurse practitioners

**Figure 2:**
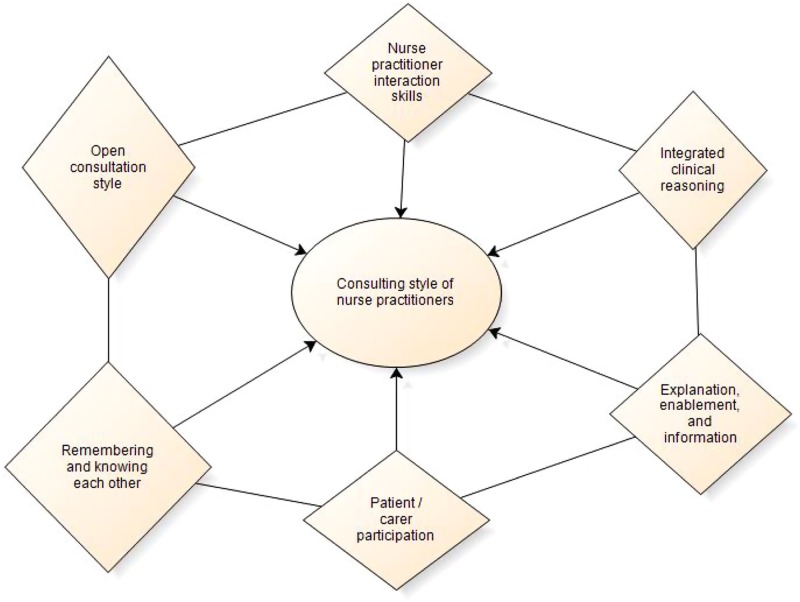
Sub-themes of the theme Consulting Style of Nurse Practitioners

‘*Consulting style of nurse practitioner*s’ was the most coded theme to emerge from the qualitative data analysis process, which enabled some of the discrete features of the communication processes and styles of interaction occurring in nurse practitioner consultations to be elaborated. Accordingly, this theme was further scrutinised to identify sub-themes to enable the concept of *Consulting style of nurse practitioners* to be fully explored, leading to the identification of six sub-themes explicating the content of the theme of *Consulting style of nurse practitioners,* which are summarily presented in Figure 2.

#### Patient/carer participation

The sub-theme of ‘*Patient*/*carer participation*’ represents the processes and opportunities for patient participation that were perceived to exist in the nurse practitioner consultations. Many patients expressed the sense that talking with the nurse practitioner was easier and more relaxed, like conversing with a friend, which was in contrast to the more formalised problem-focused interactions that may occur when consulting with a GP. For example patient 1.3 when comparing consulting with a nurse practitioner and a GP commented:

*I mean I find them quite pleasant [the nurse practitioners], with a doctor they tend to be a bit more official … {Patient 1.3}*

In further relation to this idea of friendliness and thus creating opportunities for participation, patient 3.5 said:

*They talk with you rather than down at you. In a way I suppose it’s like talking with a friend. {Patient 3.5}*

#### Integrated clinical reasoning

The sub-theme of ‘*Integrated clinical reasoning*’ relates to all three nurse practitioners verbalising their clinical reasoning to the patients/carers, and also provided information on what they saw, felt, or heard during the physical examinations of the patients. Clinical reasoning is a context-dependent way of thinking in professional health care practice, which is used to guide practice actions for determining the nature of patients’ presenting medical problems (Simmons, 2010; Barratt, 2018).

Nurse practitioner 2, elaborating on the importance of explaining clinical reasoning in her consultations said:

*So I do think it’s really important to [explain clinical reasoning], for most patients, an intelligent person who can go with you, I’ll try and say, ‘Look, this is what my thought process is about what I think is wrong … it might not be right, but this is what I think is the most likely thing … so we are going to have a trial of treatment, we might do some investigations, and then we’re going to follow it on’. {Nurse practitioner 2}*

Expounding further on verbalised clinical reasoning nurse practitioner 3 said that she observed patients felt reassured by an overt discussion of clinical reasoning as they can then question the basis for clinical decisions, such diagnostic decision making.

#### Nurse practitioner interaction skills

The sub-theme of ‘*Nurse practitioner interaction skills*’ comprised a mix of attributes which were used by the nurse practitioners in the study to successfully manage the complexities of consultation communication, and to encourage patients/carers to provide fulsome accounts of their presenting problems and associated concerns. For example, patient 1.10 noted nurse practitioner 1 helped her to articulate what she wanted to say when she was struggling to do so herself:

*Our communication was excellent. She was able to pick up on things I was trying to say when I was not very articulate. {Patient 1.10}*

Patient 3.5 noted if a patient had to be told they had not done something correctly, for example following medication usage instructions, they were advised about this in a ‘nice’ way:

*If we need to be told off, they will tell you off. They do it in such a nice way. {Patient 3.5}*

Patient 2.2 noted the nurse practitioners used conversation skills to relax him at the opening of a consultation and that they then subsequently had a two-way conversation which also helped him reveal things he wouldn’t have said otherwise:

*…they seem to calm you down and talk to you. I say something to them that I really wouldn’t thought I would have said [sic]… because they relax you first and you have [a] two-way conversation. {Patient 2.2}*

#### Explanation, enablement and information

The sub-theme of ‘*Explanation, enablement, and information*’ represents the sense conveyed in the interviews that the nurse practitioners clearly and coherently explained medical problems and treatments to patients, supported those explanations with relevant verbal and written information, which in turn enabled the patients to self-manage their medical problems. For example, patient 2.2 noted medical queries and related questions were answered in a clear non-medical style:

*They don’t tell you mumbo jumbo language. If you ask a question you get a reasonable answer that even I can understand, rather than in doctor’s language. {Patient 2.2}*

Patients commented that they clearly understood what they were supposed to do in terms of care and treatment after seeing the nurse practitioners and felt the clear explanations they received from the nurse practitioners were very important to aid their understandings. Nurse practitioner 3 noted these explanations are often supported with the provision of information, which enables patients/carers to make informed decisions about their care:

*I always say [to patients] ‘I’m not here to tell you what to do, I’ll give you the information and we can make an informed decision. {Nurse practitioner 3}*

#### Open consultation style

The sub-theme of ‘*Open consultation style*’ refers to the openness of the nurse practitioner consultations in this study. A large component of this open style is the space the patients/carers were given by the nurse practitioners to allow them to raise multiple agendas, which is in contrast to the unvoiced agendas that may sometimes occur in general practitioner consultations (Barry *et al*., 2000). For example, patient 1.10 in response to being asked in her interview about raising a second agenda item in her consultation said:

*Yes, I did. I felt a bit guilty about that really, but she did not mind at all. {Patient 1.10}*

What were the nurse practitioners’ views on multiple agenda items? Nurse practitioner 1 in response to being asked about patient 1.10 raising a second agenda item she said it did sometimes cause difficulties for her and so she tries to prioritise problems:

*…that is difficult sometimes. I guess I do have difficulty with that sometimes. I guess it is kind of prioritising, I suppose what is the most important for them, because you can’t always deal with everything. {Nurse practitioner 1}*

Nurse practitioner 1 went on to say that whilst multiple agenda items were difficult to deal with she likes patients to:

*…think that they go away feeling that they’ve got things sorted or that they have got options [to get their other problems sorted as well]. {Nurse practitioner 1}*

Nurse practitioner 3 also commented that it is difficult dealing with multiple agendas and that she too tried to prioritise patients’ presenting problems.

#### Remembering and knowing each other

The sub-theme of ‘*Remembering and knowing each other*’ relates to firstly, the nurse practitioners often remembering and commenting on patients’ previous attendances at the beginning of consultations, and secondly, the nurse practitioners and patients/carers in many, though not all instances, knowing each other as they had consulted together on numerous previous occasions.

Patient 1.10 felt that patient involvement in a consultation was related to the clinician knowing the patient:

*I think the issue about participation is to do with when they [the nurse practitioners] know you as well. It is about knowing the patient. {Patient 1.10}*

In symmetry with the patients/carers, it was also expressed by the nurse practitioners that they too had familiarity with the patients and their families. This was often because they had known the patients for extended periods of time, which meant they were familiar with their family backgrounds, and that they could then make decisions about whether or not to use such information in the consultations.

### Nurse practitioner – GP comparisons

In overview, the theme of *Nurse practitioner – GP comparisons* comprises the role differences between nurse practitioners and GPs noted by participants in the study. A prominent feature of this theme was that many patient/carer participants expected the nurse practitioners to be dealing with more minor or ‘general’ problems, whilst they would expect to see a GP for more ‘serious’ problems. For example patient 1.5, who saw nurse practitioner 1 for an acute ear infection said:

*I think, for general problems I think it [consulting with a nurse practitioner] is a very good idea. I think if I actually felt I had something more serious, I think I would rather see a doctor. But I think, for general things, I think it is absolutely fine… {Patient 1.5}*

However, the nurse practitioner participants questioned what was actually meant by ‘serious’, and cited examples where they had dealt with more complex presenting problems. It was also noted that not all patients may recognise this distinction as they just want to see a competent clinician who can provide a coherent answer to their presenting problem. It was also observed that nurse practitioners and doctors have different education and knowledge bases, which may subsequently impact on how they are expected to practice by patients, and also actually how they apply their education and knowledge in consultations. Conversely it was noted it can be difficult to make general comparative distinctions between nurse practitioners and doctors as such comparisons are not dependent on their actual clinical roles, but instead relate to their communication skills and personalities.

### Lifeworld content or lifeworld style

The theme of *Lifeworld content or lifeworld style* provides clear evidence of the presence of the everyday lifeworld in many of the observed consultations, with patients/carers feeling comfortable speaking about everyday lifeworld issues, and the nurse practitioners responding positively by encouraging the inclusion of such information. Many, though not all the patients, expressed a view that its presence was beneficial. For example, patient 3.10 said:

*I think that sort of conversation [lifeworld discussion] helps with an illness anyway…if someone is worrying about something that’s happening within their family…if you speak about it, it’s half the problem gone and they [the nurse practitioners] listen. {Patient 3.10}*

Patient 2.9 said she too would feel happy discussing lifeworld issues with a nurse practitioner, but wouldn’t feel happy to do so with a GP as she would expect a GP to be more focused on medical matters rather than the everyday of the lifeworld:

*Well, I would feel ok [discussing lifeworld issues with a nurse practitioner]. I would probably not feel the same about the GP, about doing that with the GP … probably I expect them [GPs] to be more on the medical side…rather than on the everyday side. {Mother of child patient 2.9}*

Patients 2.8 and 3.6 felt lifeworld issues should only be discussed if they are relevant to the reason for attending for a consultation. For example, patient 2.8 commented on discussing liferworld issues:

*I would only do it if it was relevant to the reason I was going in for, otherwise I would probably not waste their time on things that were not valid. {Patient 2.8}*

Conversely, two of the patients were vehement that lifeworld discussions should not be part of a clinical consultation. For example, this view was clearly expressed by patient 1.10:

*I would not involve them [the nurse practitioners in lifeworld discussions] because I believe their role is to be clinical. {Patient 1.10}*

### Nurse practitioner role ambiguity

*Nurse practitioner role ambiguity* represents the ongoing perceptual uncertainty existent amongst some patients/carers and the nurse practitioner themselves regarding the precise function and status of the nurse practitioner role. However, it must be acknowledged all the patient/carer interview participants had at least a vague understanding of the nurse practitioner role, but this understanding was not as concrete as their intrinsic, enduring understanding of a doctor’s role. This sense of ambiguity may also have been reflected in many of the patients’/carers’ previously discussed perceptions that they should see a nurse practitioner for ‘minor’ medical problems and a GP for ‘serious’ medical problems, which would indicate they perceived a boundary or ceiling existed to the plausible extent of the nurse practitioners’ clinical role capabilities.

An example of the perceived ambiguity of the nurse practitioner role was provided by patient 1.10, when she said she was uncertain about whether to see a nurse practitioner or GP for different medical problems, which also links to previously noted clinician role demarcation for minor versus serious illness noted in the nurse practitioner–GP comparisons coding node:

*I am still unsure when I would ask for a doctor and when I would ask for a health care assistant [sic] [nurse practitioner]. It is difficult to judge how ill you are and what sort of diagnosis you are looking for. {Patient 1.10}*

It must be noted patient 1.10 referred to the nurse practitioner as a ‘health care assistant’, which she did on three occasions in her interview even though she was actually talking about the nurse practitioner. This is perhaps an illustration of her perceptual uncertainty of meaning of the nurse practitioner role.

The mother of child patient 2.9 also expressed a sense of vagueness about the precise nature of the nurse practitioner role when first asked about the differences between a nurse practitioner and a GP:

*Well probably I just have a vague idea; I haven’t got any clear idea. {Mother of child patient 2.9}*

### Creating the impression of time

The theme of ‘*Creating the impression of time*’ arises from the sense conveyed by many patients/carers in their interviews that they felt the nurse practitioner had more time available to see them and that they did not feel ‘rushed’ when consulting with one of the nurse practitioners. This sense of increased time in turn led to more detailed consultation discussions occurring, which the patients/carers felt were more related to their agendas.

For example, patient 3.5 observed the nurse practitioner were very good at conveying the impression they had time to see her:

*…they’re very good at giving you the impression they have all the time in the world for you…they don’t rush you out…they’re quite prepared to sit and talk to you. {Patient 3.5}*

Nurse practitioner 1 then elucidated the perceived benefits of creating the impression of having time for patients:

*…the patient is going to feel that they’ve got what they wanted, or they have managed to say, you know talk about their problems. {Nurse practitioner 1}*

It is important to recognise this theme is more about the nurse practitioners creating the impression of time rather than them actually having extended consultation times. Analysis of the consultation time lengths in the video recorded component of the study showed the median time length of 10-minute same day appointment consultations was 9.3 minutes, and for 15-minute pre-booked appointment consultations was 13.4 minutes. So the nurse practitioners were evidently adhering to the allocated appointment slot times in their consultations, yet were still managing to convey the sense of having extra time for patients and carers consulting with them.

### Expectations for safety netting

In the theme of ‘*Expectations for safety netting’*, or making post-consultation contingency plans in case the clinician is either uncertain or wrong about their initial diagnosis or selected therapy, relates to the expectations many patients/carers expressed that the nurse practitioners would seek a further opinion from a GP if needed. It also comprises the patients/carers perceived arrangements for post-consultation follow-up, and the nurse practitioners-related responses to managing clinical uncertainty. Patient 2.8 conveyed the sense the nurse practitioners would consult with a GP colleague as needed, which resultantly helped her feel confident in seeing the nurse practitioners for perceived ‘serious’ problems:

*I would probably, in the first instance; I would talk it through with them and then see from there. Because I know that they consult, I know that [Nurse Practitioner 2} and that will always consult with colleagues. {Patient 2.8}*

All the nurse practitioners commented on the link between clinical uncertainty, that is to say not being certain about what either is wrong with a patient or how to proceed with their treatment, and discussing such cases with a GP. For example, nurse practitioner 1 said:

*I usually explain that … [if] I am not happy to do or don’t know, I will send them to a GP, always. I think generally here they [the patients] know [that]. {Nurse practitioner 1}*

## Discussion

The interview findings, in the theme of *Consulting style of nurse practitioners* in the sub-theme of *Nurse practitioner interaction skills* show that the nurse practitioners’ interaction skills encompassed attributes encouraging patients to speak in a two-way conversation, rather than their consultations being history taking sessions solely focused on nurse practitioner question-asking and patient-provided answers. These attributes included: a combined usage of verbal and non-verbal communication styles facilitating a sense of personal interest in their patients, including the application of active listening skills which encourage patients to make revelatory comments; and a recognition that focusing on communication strategies in consultations, or more simply how things are done, as opposed to emphasising the application of medical knowledge, is key to promoting patient-focused consultations whereby patients feel comfortable to express what they actually want to say and to ask questions. Such communication strategies have been characterised in previous exemplars of nurse practitioner practice as ‘healing begins with listening’, in which patient assessments are more attuned to patients relating what is going on in their everyday lifeworlds, with the nurse practitioner asking for clarifications as needed, instead of using interrogative interaction styles (Brykczynski, 2012, p. 559).

The process of *Integrated clinical reasoning*, noted as a sub-theme emerging from the interviews in the theme of *Consulting style of nurse practitioners* is a process used by all three nurse practitioners to verbalise their cognitive clinical reasoning to the patients and carers. Similar evidence of integrated clinical reasoning exists in prior studies of nurse practitioner interactions in consultations such as Paniagua (2011) where nurse practitioners thought aloud about their clinical reasoning, and also Brykczynski (1989) where nurse practitioners shared their clinical uncertainties with patients. The benefits of overt clinical reasoning being an integral part of the consultation interactions were seen by the nurse practitioners in this current study in the ‘*Integrated clinical reasoning*’ theme as facilitating an improvement in patient/carer understanding of the imprecise nature of differential diagnoses that may be discussed with them, and also enhanced reassurance regarding their medical conditions and treatment plans.

Many of the patient, carer and nurse practitioner interviewees viewed the inclusion of everyday lifeworld information in their consultations as being a positive feature of communication within their consultations. However, not all patients and carers were of the same opinion and accordingly minimised the inclusion of everyday lifeworld information in their consultations. Patients, carers and nurse practitioners perceived the interaction styles used in their consultations as facilitating opportunities for their active participation, underpinned by clear explanatory communication, and patients and carers had a sense of being listened to, and consequently felt their concerns were being directly addressed.

Many, though not all patients and carers in the study, had an ambiguous perception of the nurse practitioner role, as they were not quite clear whether nurse practitioners were functioning at a level a nurse would normally be expected to work at, or whether they function at a similar level to that of a medical doctor. The reported preference for seeing doctors when presenting with a more serious medical problem was also similarly expressed in Redsell *et al.’s* (2007b) study of the perceived differences between nurses and general practitioners, because doctors were seen by patients as having a higher level of knowledge and clinical judgement than that of nurses.

In this study, patients and carers have also reported a sense of having more time to consult with nurse practitioners, and the nurse practitioners emphasised the importance of creating an impression of having time when consulting with patients, even when they themselves felt time constrained, which corresponds with the findings of Williams and Jones (2006) identification of the expanded time factor evident in patients’ view of consulting with a nurse practitioner. Conversely this finding is in contention to the findings of previous studies of nurse practitioner consultations suggesting that increased consultation time lengths for nurse practitioners are associated with positive patient evaluations (Kinnersley *et al*., 2000; Laurant *et al*., 2005; Seale *et al*., 2005, 2006), because the nurse practitioners in this current study did not quantifiably have extended consultation times.

### Implications of the study

The implications for health care policy from this study arise from the perceived ambiguity of the nurse practitioner role amongst patients and carers which was elicited in the interviews. This perceived ambiguity existed despite the sampled clinic taking overt steps to identify itself to its patients as a nurse practitioner-led service supported with clear information about the nurse practitioner role being available in the clinic. One possible way of addressing this perceived role ambiguity would be to regulate the nurse practitioner role as a discrete part of the professional register for nurses signifying acquisition of advanced practice competencies, which would create regulatory convergence with the discrete regulation of medical consultants and general practitioners (General Medical Council, 2018). Additionally, the Royal College of Nursing (RCN) could have a statutory role in the credentialing of nurse practitioners, on a similar basis to the medical Royal Colleges in the UK who credential specialist doctors with statutory links to the General Medical Council’s register (Academy of Medical Royal Colleges, 2018). Currently, the RCN can only offer voluntary credentialing of advanced level nursing practice without any links to the Nursing and Midwifery Council’s register, as there is no statutory imperative to enable that formal linkage process (RCN Professional Services, 2017).

In relation to practice development, this study demonstrates the importance of clinicians being able to convey to patients and carers a sense of having time for them in their consultations, without correspondingly extending consultation time lengths. Furthermore, this study highlights the benefits of clinicians verbally sharing their thoughts on clinical reasoning in consultations, and communicating in a personable everyday lifeworld style with patients and carers. Thus education programmes for clinicians working in advanced practice roles should emphasise the importance of openly sharing clinical reasoning with patients, which can enable shared decision making (Coulter and Collins, 2011; Department of Health, 2012).

## Limitations

An area of limitation is the time length of some of the patient/carer interviews. The mean time duration of the three nurse practitioner interviews was 41.8 minutes, whilst the mean time duration of the 11 patient/carer interviews was much shorter at 9.6 minutes. Additionally, all the nurse practitioner interviews were face-to-face interviews whilst the patient/carer interviews were a mix of face-to-face and telephone interviews. It was initially intended to conduct all the patient/carer interviews as face-to-face interviews, but when facing the realities of recruitment to the study the flexibility of offering telephone interviews ensured recruitment of a sufficient number of participants for the interview component of the study. The five face-to-face interviews with patients had longer time ranges of 10–15 minutes and subsequently elicited more information than the six telephone interviews with patients/carers, which had shorter time ranges of 5–10 minutes. Looking at these shorter time durations for the patient/carer interviews, particularly so for the telephone interviews, it could be argued that those time lengths were not long enough to generate sufficient data in the interviews. However, interesting data was generated across the patient/carer interviews, albeit more in-depth in the face-to-face interviews. Given that the patients were reflecting on a brief 10- to 15-minute consultation experience, it is not so surprising the interviews were quite short.

In relation to rigour the member checking process led both the researcher and nurse practitioner participants to reflect on their research experiences. However, logistical constraints meant it was not possible to similarly follow-up the patient and carer interview participants, which would have been potentially beneficial for further developing the study’s findings.

## Conclusion

This study has complemented the findings of other studies of nurse practitioner consultation communication, which all commonly identify the presence and importance of patient-centred, lifeworld style interactions in nurse practitioner consultations, by conjointly examining patients’, carers’ and nurse practitioners’ perceptions of those consultations. From the overview literature searching conducted for the overall case study, it is evident a meta-synthesis of qualitative research in this area of inquiry still needs to be completed (Barratt, 2016). Accordingly, it is proposed that a meta-synthesis of available qualitative research on nurse practitioner consultation communication be completed, in order to further understand the interactive nature of communication in nurse practitioner consultations. The meta-synthesis would particularly be looking for recurring themes and inductive theories emerging from the body of qualitative research regarding nurse practitioner consultations that would facilitate a deeper understanding of nurse practitioner communication in clinical consultations for enhancing social interactions with patients and carers, which in turn may potentially optimise consultation outcomes, such as increased patient satisfaction and enablement (Barratt, 2018).
